# CoNVaQ: a web tool for copy number variation-based association studies

**DOI:** 10.1186/s12864-018-4732-8

**Published:** 2018-05-18

**Authors:** Simon Jonas Larsen, Luisa Matos do Canto, Silvia Regina Rogatto, Jan Baumbach

**Affiliations:** 1 Department of Mathematics and Computer Science, University of Southern Denmark, Campusvej 55, Odense, DK-5230 DK Denmark; 2 Chair of Experimental Bioinformatics, Wissenschaftszentrum Weihenstephan, Technical University of Munich, Maximus-von-Imhof-Forum 3, Freising-Weihenstephan, 85354 DE Germany; 3 Department of Climical Genetics, Vejle Hospital and Institute of Regional Health Research, University of Southern Denmark, Beriderbakken 4, Vejle, DK-7100 DK Denmark; 4 International Center for Research (CIPE), A.C. Camargo Cancer Center, Tagua 440, Sao Paulo, 01508-010 BR Brazil

**Keywords:** Copy number variation, Association studies

## Abstract

**Background:**

Copy number variations (CNVs) are large segments of the genome that are duplicated or deleted. Structural variations in the genome have been linked to many complex diseases. Similar to how genome-wide association studies (GWAS) have helped discover single-nucleotide polymorphisms linked to disease phenotypes, the extension of GWAS to CNVs has aided the discovery of structural variants associated with human traits and diseases.

**Results:**

We present CoNVaQ, an easy-to-use web-based tool for CNV-based association studies. The web service allows users to upload two sets of CNV segments and search for genomic regions where the occurrence of CNVs is significantly associated with the phenotype. CoNVaQ provides two models: a simple statistical model using Fisher’s exact test and a novel query-based model matching regions to user-defined queries. For each region, the method computes a global q-value statistic by repeated permutation of samples among the populations. We demonstrate our platform by using it to analyze a data set of HPV-positive and HPV-negative penile cancer patients.

**Conclusions:**

CoNVaQ provides a simple workflow for performing CNV-based association studies. It is made available as a web platform in order to provide a user-friendly workflow for biologists and clinicians to carry out CNV data analysis without installing any software. Through the web interface, users are also able to analyze their results to find overrepresented GO terms and pathways. In addition, our method is also available as a package for the R programming language. CoNVaQ is available at https://convaq.compbio.sdu.dk.

## Background

Copy number variation (CNV) is a type of structural variation in the genome in which a large segment of the DNA is either duplicated or deleted. Genome-wide association studies (GWAS) have been an important tool for discovering associations between genomic variants and disease phenotypes. GWAS data analysis methods have generally focused on single-nucleotide polymorphisms (SNPs) but can be applied to CNVs as well in order to determine the impact of larger structural variations on traits or phenotypes. Recent studies have shown that a large number of CNVs are present in healthy individuals, and are a significant source of genetic diversity in the population [1]. Currently, the Database of Genomic Variants reports more than half a million CNVs with most variations ranging from 1 kb to 10 kb in size [2].

Copy number variations affecting individual genes have been linked to the susceptibility of HIV/AIDS [3], risk to develop psoriasis [4], and autism spectrum disorders [5], amongst others. CNVs have also been shown to influence gene expression [6, 7]. Functional genomic alterations may contribute to the development and progression of diseases [8]. Thus, measuring copy numbers of such genes alongside their expression may potentially also improve diagnostics.

Due to the high number of CNVs observed in healthy individuals and the rarity of disease-associated CNVs observed even in individuals with the disease, sophisticated methods are necessary in order to detect statistically significant CNVs. A large number of tools for calling CNVs from microarray and next-generation sequencing data currently exists [9, 10]. However, few methods are available for identifying CNVs associated with a phenotype. CNVRuler [11] is a graphical desktop application that builds CNV regions using one of three models: overlapping regions, reciprocal overlap and segmentation at CNV boundaries. Associations between CNV regions and phenotype are determined using either Fisher’s exact test, a chi-squared test, linear regression or logistic regression. ParseCNV [12] is a suite of command line tools for CNV-based association studies. It performs significance testing on probe-level using Fisher’s exact test. Probes in close proximity with similar *p*-values are then merged into CNVRs. To our knowledge, no web service and no query-based methods exist yet.

## Implementation

**Fig. 1:**
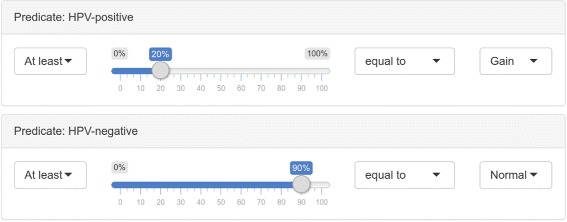
Example of a query specified through the web interface for the query-based model. This query specifies that the method should look for regions where at least 20% of the samples in the positive group have a gain in copy numbers and at least 90% of the samples in the negative group have no variation

**Fig. 2:**
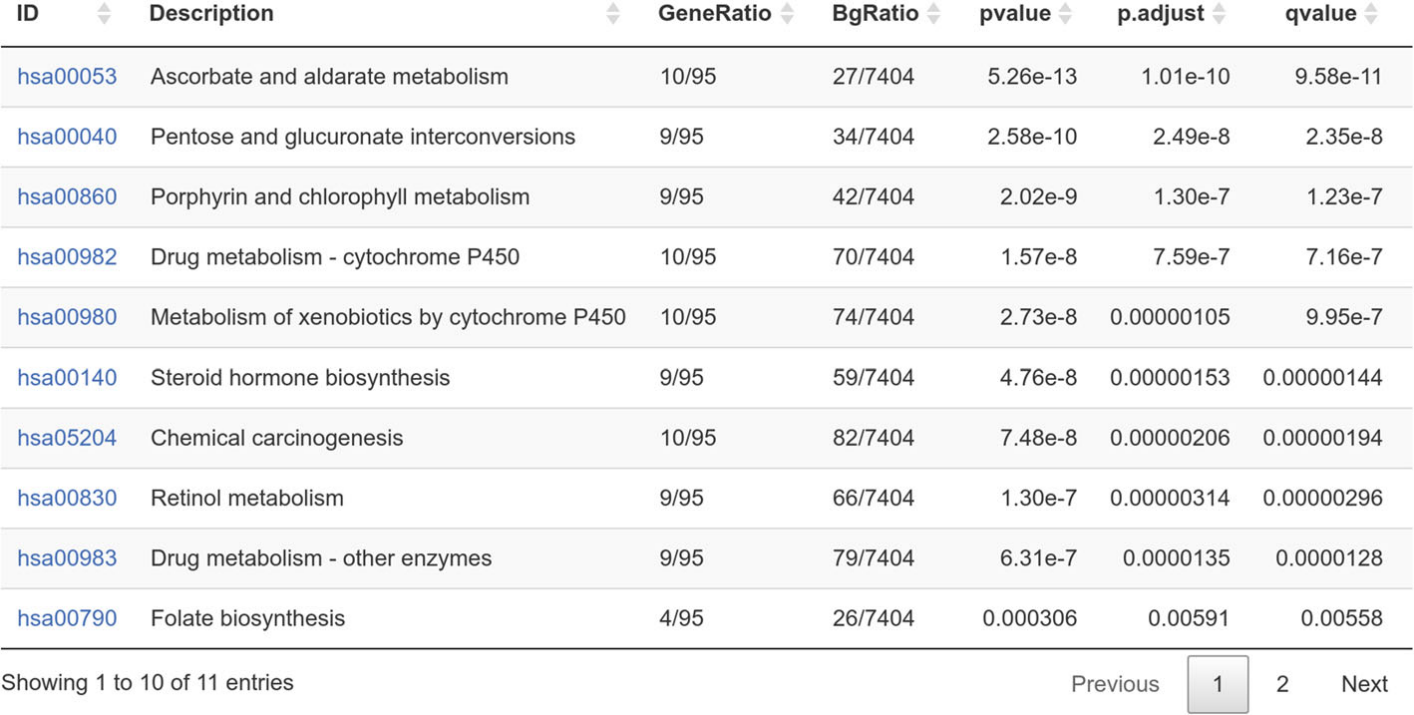
Example of output from gene set enrichment. The table shows KEGG pathways that are significantly enriched with the genes from a set of CNV regions

In this work we present CoNVAQ, a new web-based tool for copy number variation-based assocation study data analysis. Our method allows users to upload two sets of segmented CNVs (e.g. disease and healthy groups) and search for CNV regions where the occurence of CNVs is significantly associated with the classification of the samples (phenotype). Our software provides two models for signifiance testing. The first model is a traditional statistical model using Fisher’s exact test for testing significance of associations between CNV and phenotype similar to what is implemented in previous methods. The second model is a novel query-based model, that allows users to specify what patterns are considered significant using simple queries through the web interface (Fig. 1). While not as statistically robust, the second model is able to capture patterns that may not show up using a statistical hypothesis test, and is, in our opinion, easier to understand and interpret. For each CNV region found, our method computes an empirical q-value by repeated permutation of the samples between the two groups, in order to estimate significance on a genome-wide scale. Users are able to inspect the individual reported regions to obtain a distribution of events and examine in which samples a variation is observed. Our web tool also provides a gene set enrichment analysis allowing users to search for an overrepresentation of Gene Ontology (GO) terms, KEGG and Reactome pathways or disease associations (Fig. 2) among the genes located in the discovered CNV regions. Our web server works on segmented CNV calls and does not produce CNV calls from raw data, which is left to one of the many existing tools. Hence, our tool can be applied to any CNV data set regardless of the technology used. CoNVaQ is an easy-to-use web tool, where all results are computed remotely on our servers, making it usable from any desktop PC with a web browser installed. Furthermore, we also provide CoNVaQ as a package for the R programming language, allowing researchers to run analyses locally.

### Copy number variation region definition

**Fig. 3:**
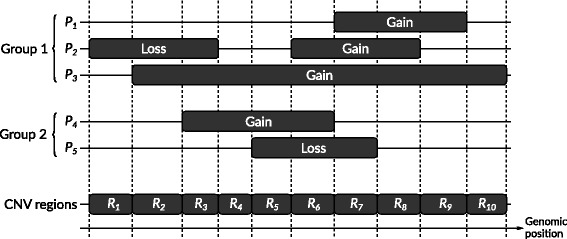
Example of chromosome segmented into ten CNV regions by CNVs from five patients. A new region is started whenever a segment from any patient starts or ends

We define a copy number variation region (CNVR) as a genomic region within a single chromosome wherein no sample changes state. Each chromosome is initially segmented into regions such that a new region starts at every end point (start or end) for every CNV among all samples (Fig. 3). As a result of this, no sample changes state within a region – only at region boundaries. Furthermore no two adjacent regions will be identical because at least one sample must change state in order for a new region to start, but two adjacent regions may have the same distribution of CNVs.

### Statistical model

The statistical model uses Fisher’s exact test for computing the significance of association between two groups of samples for some CNV event. The method considers each type of event (i.e. loss, gain and LOH) separately and classifies each sample as either having a variation of that type in the region or not. For each CNVR a 2×2 contingency table is built and a *p*-value is computed. Any region with a *p*-value less than the user-specified threshold will be reported along with the observed event.

### Query-based model

The query-based model works by extracting all regions matching some user-specified query. The user must specify a predicate for the two groups of samples being compared. Then, regions where both groups match their respective predicate are identified and reported as part of the result.

**Fig. 4:**
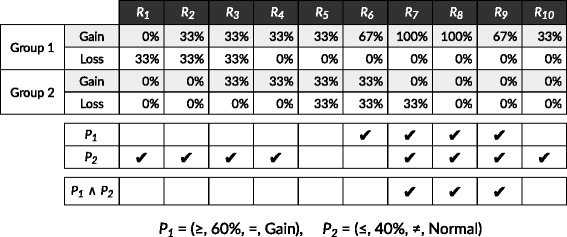
Example of matching query to CNV regions. The query ((≥,0.60,=,gain),(≤,0.40,≠,normal)) is evaluated against the ten CNV regions generated in Fig. 3. LOH frequencies have been left out for simplicity. Four regions match the predicate for group 1 (*R*_6_- *R*_9_), and eight regions match the predicate for group 2 (*R*_1_- *R*_4_, *R*_7_- *R*_10_). Three regions match the full query (*R*_7_, *R*_8_ and *R*_9_)

A predicate is defined as a tuple (*I,R,E,T*), where *I*∈{≤,≥}, *R*∈[0,1], *E*∈{=,≠} and *T*∈{Normal,Gain,Loss,LOH}. A query *Q* is defined by a pair of predicates *Q*=(*P*_1_, *P*_2_). An example of such a query could be *P*_1_=(≥,0.2,=,Gain) and *P*_2_=(≤,0.1,≠,Normal), describing that all regions in which at least 20% of samples in the first group have a gain in copy numbers while at most 10% of samples in the second group may have any kind of CNV, regardless of type, are considered significant. An example is illustrated in Fig. 4.

### Q-value computation

Empirical q-values are computed for each reported CNVR by repeatedly perturbing the distribution of samples among the two populations. Samples are distributed among the two populations such that the original population sizes are preserved. For each of the found CNVRs we compute how often we see a CNVR that is equally or more significant in each of the repetitions when performing the same query. A region is considered more significant if it spans a larger number of base pairs. This is based on the following reasoning: The null hypothesis is that the occurrence of each CNV is independent of the phenotype of the sample. Under this hypothesis, larger regions of overlapping CNVs are less likely to occur by chance than smaller regions (under the assumption that most of the genome does not exhibit any variation).

Only CNVRs of the same type are compared, i.e. finding a larger duplication will not affect the q-value of a deleted region. The position, length and type of each CNV is preserved under the perturbation. This preservation is important because we cannot reasonably assume the positions in which CNVs appear in the genome are random. By redistributing the samples among the populations while preserving their size, we instead compute the probability of observing a given overlap if there is no contingency between phenotype and the classification of samples.

### Merging adjacent CNVRs

In some cases the CNV calling method might detect two or more CNVRs in very close proximity, separated only by a small number of base pairs. Such regions may correspond to just one region with some internal variation. Furthermore, when segmenting the genome into CNV regions as described above, we may produce several regions in a row with very similar variation distributions. In order to consider such regions as singular CNVRs, CoNVaQ includes an option to merge adjacent regions within some user-specified number of base pairs. After all matching CNVRs have been selected, adjacent CNVRs of the same type that are within this threshold will be merged into a single CNVR. Regions are merged before q-values are computed, and the merging step is also performed for each repetition when computing q-values as well. For the statistical model, the *p*-value of the new region will be the largest value (least significant) of regions being merged. Regions returned from the query-based model do not have a type, meaning they can be merged with any other region if within the threshold. The frequencies of merged regions will be represented as a range, e.g. if two regions with loss of copy numbers in 23 and 31% of samples, respectively, are merged, the new region will report loss in 23-31% of samples. The length of the new region will include the gap between merged regions as well. Note that merged regions do not match the previous definition of CNVRs as each sample is no longer guaranteed to have the same state for the entire span of the region.

### Enrichment analysis

For the reported CNVRs, users are able to select one or more regions and extract all known genes overlapping those regions. A database of known genes was obtained from Ensembl [13]. A gene is said to be overlapping a CNVR if their genomic regions share at least one base pair. CoNVaQ also provides a gene set enrichment analysis. For the set of reported genes overlapping a CNVR, users can search for overrepresented Gene Ontology terms [14], KEGG pathways [15], Reactome pathways [16], Disease Ontology terms [17] and DisGeNET disease associations [18]. Enrichment analysis is carried out using the DOSE [19] and clusterProfiler [20] R packages. Statistical significance of enrichment is determined using a hypergeometric test. Let *G* be the set of genes overlapping the found CNV regions and *C* be the gene set we want to investigate for enrichment. Then a *p*-value is computed as

$$ P(X \geq k) = \sum\limits_{i=k}^{\operatorname{min}(K,n)} \frac{\binom K k \binom {N-K} {n-k}}{\binom N n}, $$

where *N* is the number of all genes, *K* is the number of genes in *C*, *n* is the number of genes in *G* and *k* is the number of genes both in *G* and *C*. Adjusted *p*-values are also computed using the Benjamini-Hochberg procedure, as well as estimated q-values using the method described in [21].

### Software requirements

CoNVaQ is implemented as a web tool accessible through a web browser. All parsing of data and computation of results is done remotely on the server, and results are then displayed in the web interface. As such, only a modern, HTML5-enabled web browser supporting Javascript is necessary in order to use CoNVaQ.

## Results

To demonstrate our platform we analyzed the penile cancer (PC) data set from [22]. It contains segmented CNV calls from 41 penile squamous cell carcinomas samples, where 14 samples were identified as HPV-positive and the remaining 27 as HPV-negative. We performed an association study between HPV-positive vs. HPV-negative samples in order to identify genomic variations that were more common in the HPV-positive group, using the two models implemented in CoNVaQ.

### Statistical model

We first searched for significant CNVs using the statistical model. The statistical model uses Fisher’s exact test to compute *p*-values for each individual CNV. Then, q-values are computed to estimate the probability of seeing a significant CNV of this type and size over the entire genome (statistical model and q-value computation are detailed in Methods section). We used a *p*-value cutoff of ≤ 0.05 for significance and enabled merging of adjacent CNVs with a distance threshold of 0 base pairs (i.e. only directly adjacent regions are merged). The method found 16 CNV regions (CNVRs) with significant *p*-value in chromosomes 2, 3, 4, 5, 8, 9, 16, 17 and 19 (Table 1). Q-values ranged from 0.0955 to 0.8415, meaning none of the regions had a statistically significant q-value (< 0.05). The most statistically significant region was a large loss event in chromosome 4. The 16 regions found here were also previously reported in [22].

**Table 1:** CNVRs extracted from penile cancer data set using the statistical model with *p*-value ≤ 0.05

						HPV-pos.	HPV-neg.
Chr	Start	End	Type	*P*-value	Q-value	freq. (%)	freq. (%)
4	9729740	24650257	Loss	0.0341	0.0955	21.4	0-3.7
2	230554659	234415376	Loss	0.0387	0.3650	28.6-35.7	0
17	15537019	18617236	Loss	0.0341	0.4632	21.4	0-3.7
2	204245506	207036312	Loss	0.0341	0.5235	21.4	0-3.7
2	237307835	238724893	Loss	0.0387	0.6505	28.6-35.7	0-3.7
9	33911175	34589574	Gain	0.0387	0.6977	28.6	3.7
4	40058630	40957235	Loss	0.0341	0.6977	21.4	0
17	19272468	20059509	Loss	0.0341	0.7198	21.4	0
17	7496965	8209436	Loss	0.0341	0.7295	21.4	3.7
8	37606006	38160563	Gain	0.0341	0.7325	21.4	0
19	51889824	52236621	Gain	0.0341	0.7465	21.4	0
16	779112	798699	Gain	0.0387	0.7907	28.6	3.7
5	130994540	131251586	Loss	0.0341	0.8083	21.4	0
2	218474389	218676793	Loss	0.0341	0.8163	21.4	0
3	53066401	53145339	Loss	0.0350	0.8387	35.7	0
2	240601010	240608450	Loss	0.0387	0.8415	28.6	3.7

Regions are sorted by q-value. The two rightmost columns contain the frequency of variations of the type corresponding to the type of event (column 4) for the case and control groups, respectively

### Query-based model

We next also searched for significant CNVRs using the query-based model. The query-based model finds regions matching some user-specified query, and q-values are then computed using the same procedure as for the statistical model (detailed in Methods section). We define two queries *Q*_loss_ and *Q*_gain_ to search for loss and gain events, respectively. The two queries are defined as follows:

$$ \begin{array}{lll} Q_{\text{loss}} &= \quad ((\geq, 0.20, =, \text{loss}), & (\leq, 0.10, =, \text{gain})), \\ Q_{\text{gain}} &= \quad ((\geq, 0.20, =, \text{gain}), & (\leq, 0.10, =, \text{loss})). \end{array} $$

These two queries specify that we are searching for regions with at least 20% of cases (HPV-positive) and at most 10% of controls (HPV-negative) having a gain or loss, respectively. Merging of adjacent regions was enabled with a distance threshold of 0 base pairs again. The *Q*_loss_ query found 23 regions in chromosomes 2, 3, 4, 5, and 17 (Table 2). The q-values ranged from 0.0120 to 0.911. The only region with a q-value < 0.05 was a large region in chromosome 2 in which the frequency of copy number loss was between 21.4 and 35.7% for the HPV-positive samples and between 0 and 7.4% for the HPV-negative samples. The *Q*_gain_ query found 15 regions in chromosomes 6, 7, 8, 9, 16, 17, 19 and 20 (Table 3). The q-values ranged from 0.276 to 0.871, and thus none of the regions had a significant q-value.

**Table 2:** CNVRs extracted from penile cancer data set using the query-based model with the *Q*_loss_ query

				HPV-pos.	HPV-neg.
Chr	Start	End	Q-value	loss (%)	loss (%)
2	204245506	240688770	0.0120	21.4-35.7	0-7.41
4	9729740	24650257	0.2037	21.4	0
4	40058630	47037351	0.3377	21.4	0-7.41
5	140934280	147709268	0.3785	21.4-28.6	3.7-7.41
5	60380235	66930240	0.3835	21.4	3.7-7.41
5	76699391	82660330	0.3955	21.4	7.41
17	13330532	18617236	0.4525	21.4	0-7.41
5	126804776	131251586	0.4948	21.4	0-7.41
5	72910129	75996874	0.5863	21.4	7.41
17	19272468	22200000	0.6252	21.4	0-7.41
17	7262327	9931292	0.6633	21.4	0-7.41
17	567713	3136246	0.6793	21.4	7.41
3	46788991	49028973	0.6957	28.6	7.41
17	4566909	6490288	0.7335	21.4	3.7-7.41
5	108507593	110029337	0.7552	21.4-28.6	7.41
2	241709000	242951149	0.7762	21.4	3.7-7.41
3	52397990	53145339	0.8732	21.4-35.7	7.41
5	148184554	148894433	0.8770	21.4	3.7
3	14191317	14755952	0.8862	21.4	7.41
5	140011232	140459066	0.8912	21.4	3.7-7.41
5	92966197	93334626	0.8935	21.4	7.41
5	55167345	55283138	0.9103	21.4	7.41
5	122689458	122739532	0.9113	28.6	7.41

Searching for regions with a loss of copy number in at least 20% of cases and at most 10% of controls. The two rightmost columns contain the frequency of copy nuber loss for the case and control groups, respectively

**Table 3:** CNVRs extracted from penile cancer data set using the query-based model with the *Q*_gain_ query

				HPV-pos.	HPV-neg.
Chr	Start	End	Q-value	gain (%)	gain (%)
9	70091642	90376569	0.2762	21.4	7.41
9	13045258	21368309	0.4562	21.4	3.7-7.41
9	119273838	124799603	0.4885	21.4-28.6	7.41
9	33871385	37132743	0.5633	21.4-28.6	3.7-7.41
20	43179131	45592378	0.6395	21.4	7.41
6	13768413	16036680	0.6512	21.4	3.7
19	50012054	52236621	0.6655	21.4	0-7.41
9	91369725	93273440	0.7047	28.6	7.41
16	463336	1664507	0.7648	21.4-28.6	3.7-7.41
17	77682387	78774742	0.7680	21.4	7.41
6	31191394	31875972	0.7963	21.4	3.7
8	37606006	38160563	0.8532	21.4	0
20	33204027	33688992	0.8565	21.4	7.41
7	54865060	55296001	0.8600	21.4	7.41
20	42433913	42557332	0.8710	21.4	7.41

Searching for regions with at least 20% of cases having a gain and at most 10% of controls having any kind of variation. The two rightmost columns contain the frequency of copy number gain for the case and control groups, respectively

In some cases, one can tighten the thresholds in the query in order to achieve more precise results. If we use the query $Q_{\text {loss}}^{*} = ((\geq, 0.30, =, \text {loss}), (\leq, 0.05, \neq, \text {normal}))$, we instead find two smaller regions in chromosome 2 (Table 4) with q-values 0.038 and 0.047. Both regions a part of the large loss event found with *Q*_loss_, but with a stronger association to HPV-positive status.

**Table 4:** CNVRs extracted from penile cancer data set using they query-based model with the $Q_{\text {loss}}^{*}$ query

				HPV-positive		HPV-negative	
Chr	Start	End	Q-value	Gain (%)	Loss (%)	Gain (%)	Loss (%)
2	233245552	234392866	0.0382	0	35.7	0	3.7
2	237307836	238138001	0.0473	0-7.14	35.7	0	3.7

Searching for regions with a copy number loss in at least 30% of cases and any kind of variation in at most 5% of controls. Columns 5 and 6 show the frequency of copy number gain and loss for the case group, and columns 7 and 8 shows the frequency of gain and loss for the control group

## Discussion

We used CoNVaQ to search for genomic regions where the occurrence of copy number variations was significantly associated to HPV status. The statistical model found 16 CNVRs with *p*-value < 0.05. However, none of the regions were reported to have a significant q-value (< 0.05) after permutation testing. This means that for all of the found CNVRs, if the samples are randomly assigned to the two groups, we will likely see an equally large region of same type with *p*-value < 0.05. This could suggest that for these regions, further validation is necessary to determine whether they are in fact associated with the phenotype. The query-based model found 23 regions for *Q*_loss_ and 15 regions for *Q*_gain_. One region had a significant q-value, namely region in chromosome 2 with copy number loss associated with positive HPV status.

The large discrepancy between the *p*-values and q-values reported by the statistical model suggests that looking at the individual regions is not sufficient to determine whether a CNVR is indeed significantly associated to a phenotype. The cohort used in our analysis consists of only 41 samples which is evidently too few to determine significance with high confidence. It illustrates the need for doing proper permutation testing and reporting q-values along with the standard *p*-values. One factor, however, is that the q-value statistic is computed over the entire genome. If the analysis is restricted to a single chromosome, the q-values generally become much smaller. However, given that association studies generally aim to find any variation in any chromosome associated to a trait or phenotype, we believe the q-value statistic should be computed over the entire genome.

Our method currently supports only discrete labels for CNV calls. When the copy number for each CNV is discretized before analysis, information that may potentially be important is discarded. Future versions of CoNVaQ will be extended to also support numerical values for CNV calls in addition to the three categories currently supported (gain, loss and LOH). This would enable determining significance using regression analysis (e.g. linear and logistic regression) and statistical hypothesis tests such as Student’s *t*-test or the Mann-Whitney *U* test.

Our platform does not currently support uploading and processing raw genomic data. While this would improve user-friendliness, we believe this is currently out of scope for our method. For now we believe that quality control and CNV calling is best handled by the software tools provided with the CNV detection platforms. By working with processed CNV data instead, it makes our tool agnostic to the detection method used, and can thus be used with both aCGH and next-generation sequencing data.

## Conclusions

In this paper we presented CoNVaQ, a web tool for copy number variation-based association studies. CoNVaQ implements two models: a statistical model using Fisher’s exact test for significance estimation and a novel query-based model that extract CNV regions matching some user-specified query. Our method provides a secondary significance method by computing an empirical q-value by repeated random permutation of the samples among the two groups. CoNVaQ is provided as a web tool accessible online, making it very simple to use and requiring no additional software besides a web browser. Through the web interface we also provide gene set enrichment analysis to easily determine whether a set of CNV regions are associated with GO terms, molecular pathways or diseases. We used CoNVaQ to analyze a data set containing segmented CNV calls for 41 penile cancer patients categorized into HPV positive and HPV negative. While the standard statistical analysis found regions with significant *p*-value (< 0.05), no region had a significant q-value as well. Q-values were observed to generally be significantly larger than the *p*-values for the corresponding regions suggesting that looking at each region in isolation is not sufficient for determining significance. While the q-value measure appears to be conservative, we argue that a global significance measure is necessary to reduce type I errors.

## Availability and requirements

**Project name:** CoNVaQ

**Project home page:** https://convaq.compbio.sdu.dk

**Archived version:** DOI: 10.5281/zenodo.1217803 (backend), DOI: 10.5281/zenodo.1217898 (frontend).

**Operating system(s):** Platform independent

**Programming language:** R and C++

**Other requirements:** Browser supporting HTML5 and Javascript

**License:** MIT

**Any restrictions to use by non-academics:** None

## Abbreviations

CNV: Copy number variation
CNVR: Copy number variation region
GWAS: Genome-wide association study
LOH: Lack of heterozygosity
SNP: Single nucleotide polymorphism
